# Comprehensive Analysis and Co-Expression Network of mRNAs and lncRNAs in Pressure Overload-Induced Heart Failure

**DOI:** 10.3389/fgene.2019.01271

**Published:** 2019-12-12

**Authors:** Shuping Chen, Qiong Ma, Yanbo Xue, Jingwen Zhang, Guodong Yang, Tingzhong Wang, Aiqun Ma, Ling Bai

**Affiliations:** ^1^Department of Cardiovascular Medicine, First Affiliated Hospital of Xi’an Jiaotong University, Xi’an, China; ^2^Department of Cardiology, First Affiliated Hospital of Sun Yat-Sen University, Guangzhou, China; ^3^Shaanxi Key Laboratory of Molecular Cardiology, Xi'an Jiaotong University, Xi’an, China; ^4^Key Laboratory of Environment and Genes Related to Diseases (Xi’an Jiaotong University), Ministry of Education, Xi’an, China

**Keywords:** heart failure, cardiac hypertrophy, mRNA, lncRNA, expression profile, co-expression analysis, transcription factor

## Abstract

**Aim:** Heart failure (HF) is the end stage of various cardiovascular diseases. However, the precise regulation of gene expression profiles and functional mechanisms of long non-coding RNAs (lncRNAs) in HF remain to be elucidated. The present study aimed to identify the differentially expressed profiles and interaction of messenger RNAs (mRNAs) and lncRNAs in pressure overload-induced HF.

**Methods:** Male Sprague-Dawley rats were randomly divided into the HF group and the sham-operated group. HF was induced by the transverse aortic constriction (TAC) surgery. The cardiac expression profiles of mRNAs and lncRNAs in HF were investigated using the microarray. Bioinformatics analyses and co-expression network construction were performed from the RNA sequencing data.

**Results:** The expression profiles of mRNAs and lncRNAs showed significant differences between HF and controls. A total of 147 mRNAs and 162 lncRNAs were identified to be differentially expressed with a fold change of >2 in HF. The relative expression levels of several selected mRNAs and lncRNAs were validated by quantitative PCR. Gene Ontology (GO) and Kyoto Encyclopedia of Genes and Genomes (KEGG) pathway analyses indicated that diverse pathways were involved in the molecular mechanisms of cardiac hypertrophy and HF including immune response, smooth muscle contraction, ion transmembrane transport. The mRNA-lncRNA and transcription factors (TFs)-lncRNA co-expression networks were constructed and several genes and TFs were identified as key regulators in the pathogenesis of HF. Further functional prediction showed that the lncRNA NONRATT013999 was predicted to *cis-*regulate mRNA CDH11, and NONRATT027756 was predicted to *trans-*regulate HCN4.

**Conclusion:** This study revealed specific expression regulation and potential functions of mRNAs and lncRNAs in pressure overload-induced HF. These results will provide new insights into the underlying mechanisms and potential therapeutic targets for HF.

## Introduction

Heart failure (HF) is the end stage of various cardiovascular diseases, with high morbidity and mortality, leading to a significant burden on people’s health (Ponikowski et al., 2016). Diverse pathophysiologic processes are involved in the failing heart, such as cardiomyocyte hypertrophy, myocardial fibrosis, energetic metabolism, ion channel disorder, and other pathways (Munzel et al., 2015; Zile et al., 2015; Gonzalez et al., 2018; Noordali et al., 2018). Cardiac pressure overload is one of the main mechanisms of HF, which can result in cardiac hypertrophy, cardiac structural remodeling, and electrical remodeling, related to over-expression or low-expression of functional genes and gene clusters (Nomura et al., 2018). However, the precise pathogenesis and specific gene expression regulation of cardiac hypertrophy or pressure overload-induced HF are still not well elucidated.

Long non-coding RNAs (lncRNAs) are a heterogeneous group of noncoding transcripts longer than 200 nucleotides (Mercer et al., 2009). LncRNAs are found to combine other RNAs or DNAs and have complicated transcriptional or post-transcriptional regulatory functions (Mercer et al., 2009; Geisler and Coller, 2013). These RNAs have been shown to play potential roles in many physiological and pathological processes of cardiovascular diseases (Uchida and Dimmeler, 2015; Bar et al., 2016). Although several lncRNAs have been recently discovered to be associated with cardiac development and pathophysiology (Wang et al., 2014; Yuan et al., 2019b), the previous results were limited to some extent and in need of further exploration. Moreover, the roles and regulation of lncRNAs in HF are still not clear. A few current studies has investigated the lncRNA expression profiles in HF with different etiologies, including ischemic heart failure (Yang et al., 2014; Gao et al., 2015) and dilated cardiomyopathy (Li et al., 2018). While there are scarcely any comprehensive research and functional analysis of messenger RNAs (mRNAs) and lncRNAs in HF induced by pressure overload, and the underlying mechanisms and pathways remain poorly understood.

Therefore, in the current study we subjected rats to transverse aortic constriction (TAC) surgery to develop a pressure overload-induced HF model. Then we investigated the complete characteristics of myocardial mRNA and lncRNA expression profiles in pressure overload-induced HF based on microarray data, compared with controls. In addition, we performed bioinformatics analyses of differentially expressed mRNAs to explore enriched pathways involved in HF. Moreover, we constructed a mRNA-lncRNA co-expression network of the myocardial transcript profiling in HF for detailed functional prediction. We further determined several target genes and regulatory transcription factors (TFs) by *cis*- and *trans*-regulation prediction of lncRNAs. Results we got suggested that mRNAs and lncRNAs expressed abnormally in HF, and lncRNAs participated in the pathogenesis of HF, providing novel insights into the mechanisms or treatment targets underlying HF.

## Materials and Methods

### Animals

Male Sprague-Dawley (SD) rats (180–200 g), obtained from the Laboratory Animal Center of Xi’an Jiaotong University Health Science Center (Xi’an, Shaanxi, China), were randomly divided into two groups: the HF group (n=10) and the sham-operated (SO) group (n = 10). The HF group underwent a TAC surgery, and the SO group underwent a similar surgery but without the aorta ligation. All animal experiments were performed following the guidelines for the care and use of laboratory animals and approved by the Ethical Committee of Xi’an Jiaotong University.

### Pressure Overload-Induced Heart Failure Model

The pressure overload-induced HF model was induced by the TAC surgery conducted as described previously with modification (Xi et al., 2009). Briefly, the rats were anesthetized by 2% sodium pentobarbital (30–50 mg/kg, i.p.). Then the thoracic cavity was opened at the second or the third intercostal space through the right parasternal approach and the aorta was exposed. A silk suture was passed under the aorta 4–6 mm above the aortic valve. A sterile 4F catheter was placed around the ascending aorta, and the suture was fastened around the catheter and the aorta. The catheter was removed quickly, leading to a reduction of the aortic diameter to 1.32 mm. Then the thoracic cavity was closed. The SO rats were used as controls. To characterize the cardiac dimensions and function, both groups underwent echocardiographic measurements at 16 weeks after surgery. Afterward, all groups were sacrificed to harvest myocardium tissues which were snap-frozen in liquid nitrogen and stored at −80°C subsequently for microarray analysis.

### RNA Extraction and Quality Control

Myocardial total RNA was isolated from myocardium tissues from the HF and control groups using TRIzol reagent (Invitrogen, CA, USA) following manufacturer’s instruction. The concentration and purity of the RNA were measured using NanoDrop ND-1000 spectrophotometer (Thermo Scientific). The integrity of the RNA was determined using denaturing gel electrophoresis (Agilent Technologies, CA, USA).

### Microarray and Data Analysis

The Agilent Rat lncRNA+mRNA Array v1.0 was designed for profiling both mRNAs and lncRNAs of the rat genome containing a total of 30,254 rat mRNAs and 22,020 rat lncRNAs which were collected from multiple databases, including NCBI RefSeq, UCSC, Ensembl, and other literatures. The sample labeling and microarray hybridization were performed according to the CapitalBio cRNA Amplification and Labeling Kit (CapitalBio, Beijing, China). In brief, the extracted RNA was amplified and transcribed to complementary DNA (cDNA) using the CbcScript reverse transcriptase with cDNA synthesis system (CapitalBio, Beijing, China). The amplified RNAs were adopted Klenow enzyme labeling strategy using CbcScrip II reverse transcriptase. Furthermore, arrays hybridization was conducted in the Agilent Hybridization Oven. Then the hybridized mRNA+lncRNA arrays were washed and scanned using the Agilent Scanner G2505C (Agilent Technologies, CA, USA).

The array data were further analyzed using the GeneSpring software v13.0 (Agilent Technologies, CA, USA). Differentially expressed mRNAs and lncRNAs between two groups were selected with a threshold ≥2 or ≤−2 fold change (FC) and *P *< 0.05 according to previous studies (Li et al., 2018; Zhu et al., 2019). Hierarchical Clustering was carried out with average linkage to assess the differential mRNA and lncRNA expression patterns. The microarray analyses were performed by CapitalBio Technology (Beijing, China).

### qPCR Validation

In order to confirm the reliability of the reliability of the microarray data, several differentially expressed mRNAs and lncRNAs were selected for validation by quantitative real-time PCR (qPCR). All reactions were performed in triplicate. Actin was used as an internal control to normalize the data. The relative expression levels of mRNAs and lncRNAs were calculated using the 2^−ΔΔCt^ method. The specific primers used for each gene were listed as follows: NM_031601 (Cacna1g), forward CAGTCCTGGTGTCTGCCTC and reverse GTGTCTTTCTTTGGGGAGGGT; NM_019266 (Scn8a), forward CCAGAAGAACGGGAACGGAA and reverse CCAGAAGAACGGGAACGGAA; NONRATT027756, forward AATGCACAGGGGAGGTTACG and reverse CTCCCAGCC​TGTAGGTCTCT; actin, forward GTTGTCTCCTGCGACTTCA and reverse TGGTCCAGGGTTTCTTACTC.

### Gene Ontology and Kyoto Encyclopedia of Genes and Genomes Pathway Analysis

Gene Ontology (GO) and Kyoto Encyclopedia of Genes and Genomes (KEGG) pathway analyses were performed to explore the functions of these differentially expressed mRNAs. GO analysis contained biological process (BP), cellular component (CC), and molecular function (MF). Differentially expressed mRNAs were classified to different GO terms which described gene functions and attributes based on the GO database (http://www.geneontology.org). Enriched biochemical pathways were identified according to KEGG database (http://www.genome.jp/kegg/) and PANTHER Gene analysis tools (http://www.pantherdb.org). The significant *P*-value of the pathway correlated is less than 0.05.

### Construction of mRNA-lncRNA Co-Expression Network

Correlation analysis of the differentially expressed mRNAs and lncRNAs was performed to construct a mRNA-lncRNA co-expression network. For each pair of genes, the Pearson’s correlation coefficient was calculated and greater than 0.99 was considered significant. Those mRNAs and lncRNAs with significant correlation were selected for co-expression network construction using the bioinformatics software Cytoscape. The degree centrality was used to reveal the relative importance of genes in the network analysis.

### Target Prediction and Transcription Factors Prediction of lncRNAs

Regulatory mechanisms of lncRNAs can be divided into *cis*- and *trans*-regulation manner. The *cis*-regulation prediction was performed based on the mRNA-lncRNA co-expressed pairs with Pearson’s correlation coefficient >0.99 and closely related genomic loci within 10 kb. The *trans*-regulation prediction was conducted using the Standalone BLAT v.35x1 fast sequence search command line tool to compare the full sequence of lncRNAs and the 3’UTR of co-expressed mRNAs to select lncRNA-mRNA pairs with similar sequences. Furthermore, the interaction between lncRNAs and correlative TFs was analyzed to discover the potential regulatory targets of lncRNAs using Match-1.0 Public prediction tool. The TFs-lncRNA network was constructed using Cytoscape software.

### Statistical Analysis

All results were presented as mean ± SD. Statistical analysis was performed using the Student’s *t-*test for comparison between two groups. All statistics were analyzed using GraphPad Prism 8.0.2 and SPSS 22.0 software. A value of *P* < 0.05 was considered statistically significant.

## Results

### Pressure Overload-Induced Heart Failure Model

**Figure 1:**
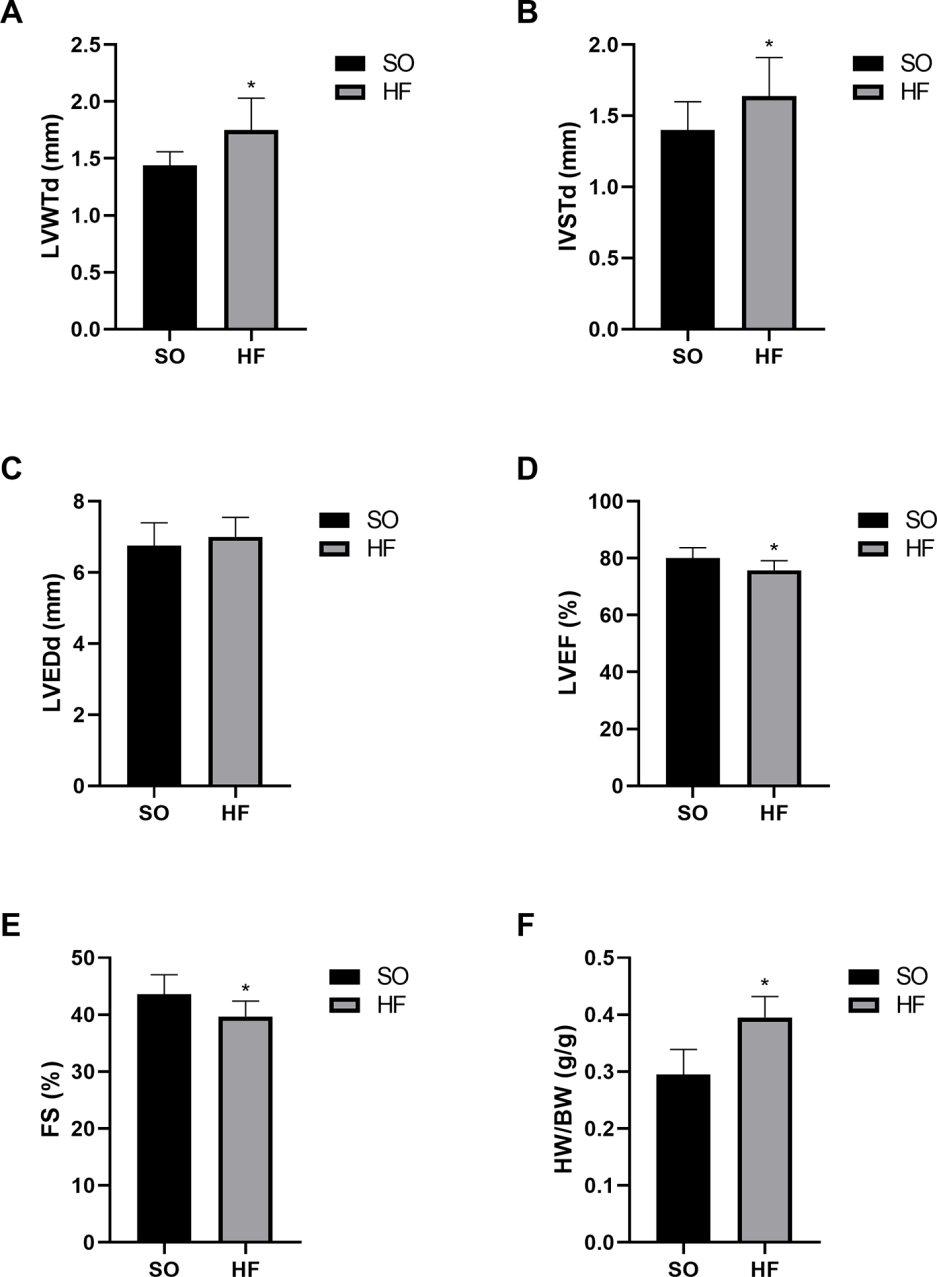
Characteristics of the sham-operated (SO) rats and pressure overload-induced HF rats at 16 weeks after transverse aortic constriction (TAC). Increased diastolic left ventricle wall thickness **(A)** and interventricular septal thickness **(B)** indicated significant left ventricular hypertrophy in TAC rats without effects on left ventricular end-diastolic diameter **(C)**. Decreased left ventricular ejection fraction **(D)** and fractional shortening **(E)** indicated significant cardiac dysfunction. Increased heart weight index **(F)** confirmed myocardial hypertrophy in TAC rats. n = 10 per group. *P < 0.05 HF *vs.* SO. SO, sham-operated; HF, heart failure; LVWTd, left ventricle wall diastolic thickness; IVSTd, interventricular septal diastolic thickness; LVEDd, left ventricular end-diastolic diameter; LVEF, left ventricular ejection fraction; FS, fractional shortening; HW/BW, heart weight/body weight ratio.

At 16 weeks after TAC surgery, the HF rats had significant left ventricular hypertrophy and systolic dysfunction compared with the SO rats based on the echocardiographic data, which indicated the presence of pressure-overload-induced HF. (**Table S1**). HF rats showed a significant increase in ventricle wall and interventricular septal thickness (**Figures 1A–C**), and appeared a significant decrease of left ventricular ejection fraction and fractional shortening (**Figures 1D, E**), compared with the SO rats (*P* < 0.05). Furthermore, the heart weight index (HWI, heart weight/body weight ratio) also increased significantly in HF rats (**Figure 1F**). In addition, our previous invasive hemodynamic data (Xi et al., 2009) demonstrated significant cardiac dysfunction in the HF model made by same methods. The plasma ANP level also increased significantly in HF rats (Xi et al., 2009), confirming the successful establishment of the pressure overload-induced HF model.

### mRNA and lncRNA Expression Profiles in Heart Failure

**Figure 2:**
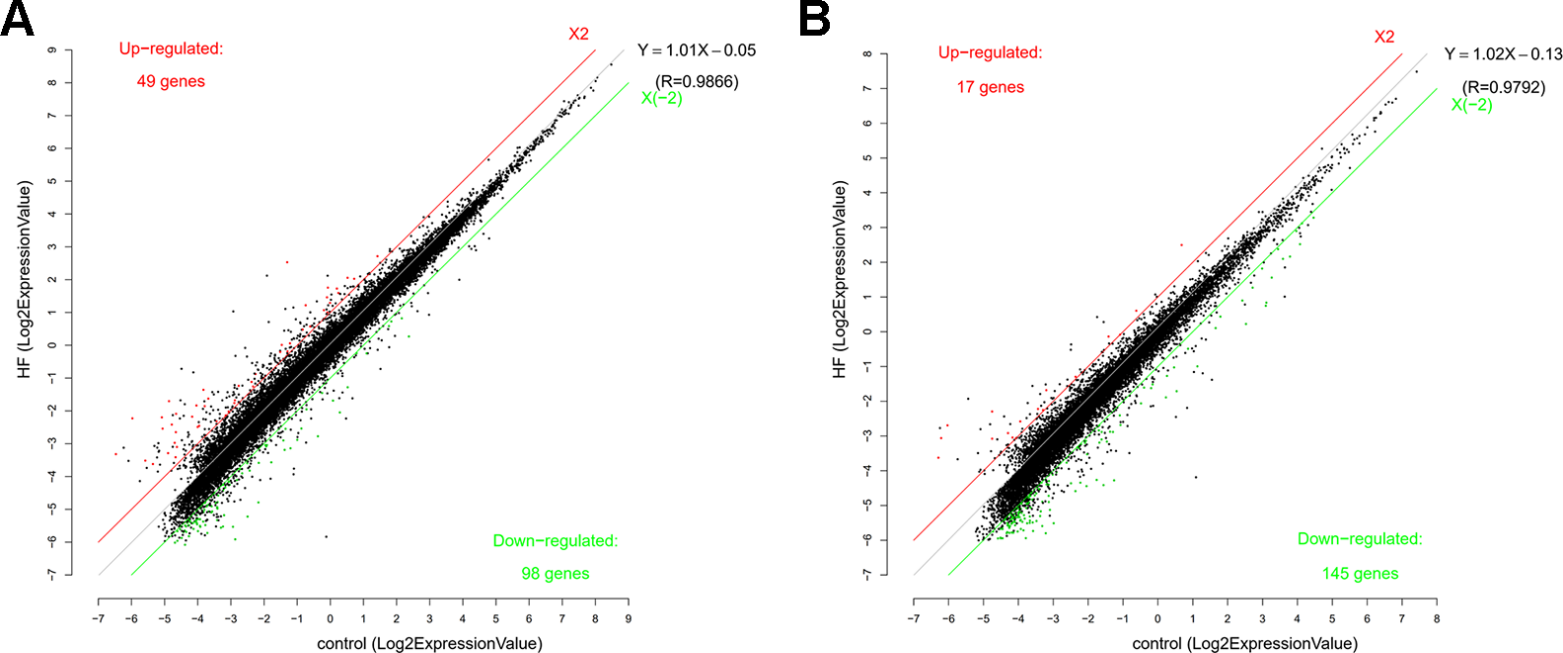
Scatter function of messenger RNA (RNA) **(A)** and long non-coding RNA (lncRNA) **(B)** expression profiles in heart failure (HF) compared with controls. The number of up-regulated and down-regulated mRNAs and lncRNAs were given in the figure. The values of the X and Y axes in the scatter plot were the averaged normalized signal values of the HF and sham-operated group (log2 scale). The red line and the green line were threshold boundary lines for up-regulated or down-regulated mRNAs and lncRNAs respectively (the significant fold change given was 2.0). The black line was the fitting line of total expression and the fitting equation was calculated. R represented the related coefficient of two group samples.

**Figure 3:**
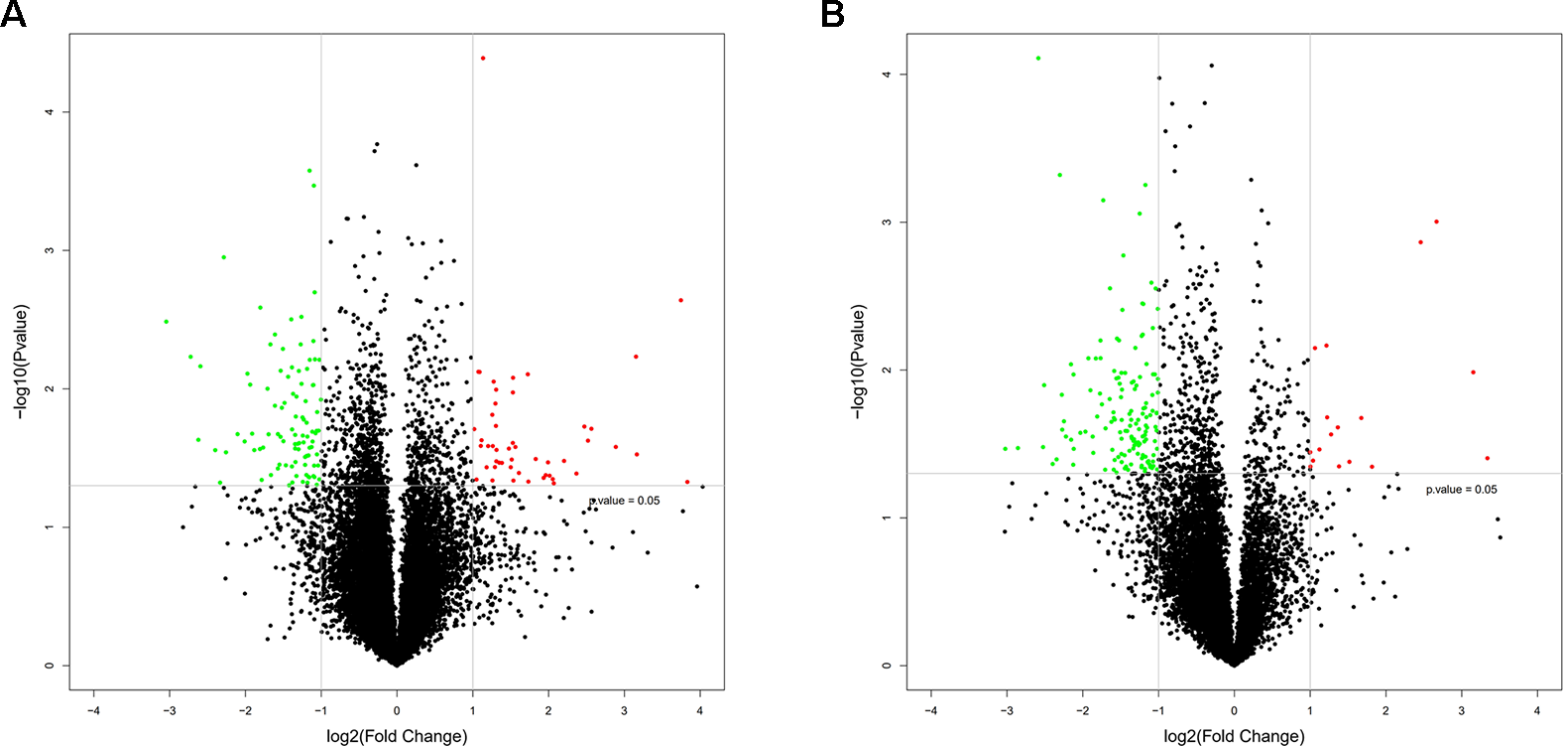
Volcano plots of messenger RNA (mRNA) **(A)** and long non-coding RNA (lncRNA) **(B)** expression profiles in heart failure compared with controls. The horizontal line represented a p-value of 0.05 in log10 scale, and the vertical lines represented up and downregulation with twofold change. The red and green points represented the up-regulated and down-regulated mRNAs and lncRNAs respectively, and the black points were no significantly differentially expressed genes.

To study the regulation of mRNAs expression and the probable role of lncRNAs in the HF rats, microarray analysis was conducted to determine the mRNA and lncRNA expression profiles of the HF rats compared with the SO rats. Furthermore, mRNA and lncRNA expression profiles were presented in scatter plots (**Figure 2**) and volcano plots (**Figure 3**) as HF group compared to SO group, according to the definition that the threshold value for the significance used to define up-regulation or down-regulation of genes was a fold change of >2, as well as with a P-value of < 0.05.

The mRNA profiling showed 147 of 22,279 mRNAs were tested to be differentially expressed according to significant levels of difference expression at least a two-fold change in the HF rats compared with the SO rats, with 49 up-regulated and 98 down-regulated respectively (**Figure 2A**, **Figure 3A**, and **Table 1**). The top 20 differentially expressed mRNAs were listed in **Table 2**. Among the dysregulated mRNA transcripts, thrombospondin 4 (Thbs4, NM_017133) was the most up-regulated, with FC 14.233, whereas LOC102555023 (XR_361242) was the most down-regulated with FC 8.253.

**Table 1:** Relative differentially expressed messenger RNAs and long non-coding RNAs in heart failure compared with controls.

	mRNA		lncRNA	
	Up-​regulated	Down-regulated	Up-​regulated	Down-regulated
Fold change 2–5	40	92	13	138
Fold change 5–10	7	6	3	7
Fold change >10	2	0	1	0
Total	49	98	17	145
Differentially expressed RNAs	147		162	

**Table 2:** Top 20 differentially expressed messenger RNAs in heart failure compared with controls.

GenBank accession	Gene symbol	Fold change	*P*-value	Regulation
NM_017133	*Thbs4*	14.233	0.047	Up
NM_001106725	*Agr2*	13.416	0.002	Up
NM_134401	*Crtac1*	8.966	0.030	Up
NM_001024742	*Naa11*	8.897	0.006	Up
XR_361242	*LOC102555023*	8.253	0.003	Down
NM_203325	*Mup5*	7.395	0.026	Up
NM_001001508	*Lppr4*	6.151	0.023	Down
NM_001113335	*Slc9a2*	6.038	0.007	Down
XM_006238223	*LOC688547*	5.916	0.019	Up
NM_012641	*Reg1a*	5.735	0.024	Up
NM_147212	*LOC259244*	5.548	0.019	Up
NM_001191822	*Rhbdl1*	5.272	0.028	Down
NM_147215	*Obp3*	5.160	0.041	Up
NM_001134641	*RGD1560884*	5.043	0.048	Down
BC088257		4.780	0.029	Down
NM_147212	*LOC259244*	4.608	0.033	Up
NM_001012011	*Lig3*	4.300	0.021	Down
NM_030840	*Slc26a5*	4.196	0.048	Up
NM_001005897	*Clec4e*	4.155	0.045	Up
NM_012508	*Atp2b2*	4.038	0.042	Up

Based on the same criteria to screen a total of 13,528 lncRNAs, 162 lncRNAs were found differentially expressed in the HF group compared to the control group. As shown in the scatter plot and volcano plot, 17 lncRNAs were up-regulated, while 145 lncRNAs were down-regulated (**Figure 2B**, **Figure 3B**, and **Table 1**). The most up-regulated and down-regulated lncRNAs were XR_344816.1 with FC 10.118 and XR_591656.1 with FC 8.127, respectively (shown in **Table 3**).

**Table 3:** Top 20 differentially expressed long non-coding RNAs in heart failure compared with controls.

Target ID	Fold change	*P*-value	Regulation	Chromosome position
XR_344816.1	10.118	0.039	Up	chr3: 1138511–1141696
XR_358422.2	8.891	0.010	Up	chr11: 2304132–2305501
XR_591656.1	8.127	0.034	Down	chr3: 1426625–1428019
NONRATT024270	7.241	0.034	Down	chr6: 2114563–2115162
XR_590356.1	6.351	0.001	Up	chr1: 3055953–3059354
XR_600156.1	6.005	0.000	Down	chr3: 1232115–1240608
NONRATT025409	5.752	0.033	Down	chr6: 407523–407990
NONRATT023839	5.695	0.013	Down	chr5: 3692775–3693535
XR_593816.1	5.495	0.001	Up	chr14: 4823160–4825467
NONRATT016350	5.269	0.043	Down	chr2: 4722257–4727845
NONRATT026008	5.084	0.040	Down	chr7: 3427462–3432042
NONRATT022872	4.932	0.000	Down	chr5: 749024–749401
NONRATT000918	4.845	0.015	Down	chr1: 3023674–3025708
NONRATT003405	4.828	0.025	Down	chr1: 1319862–1320497
XR_591425.1	4.752	0.022	Down	chr2: 114669–196442
XR_356919.2	4.666	0.028	Down	chr9: 1097060–1116238
XR_357854.2	4.457	0.009	Down	chr10: 4300924–4301531
NONRATT029506	4.451	0.030	Down	chr9: 1507295–1508007
NONRATT013850	4.365	0.044	Down	chr18: 1011665–1012798
XR_600157.1	4.354	0.011	Down	chr3: 1232115–1235264

**Figure 4:**
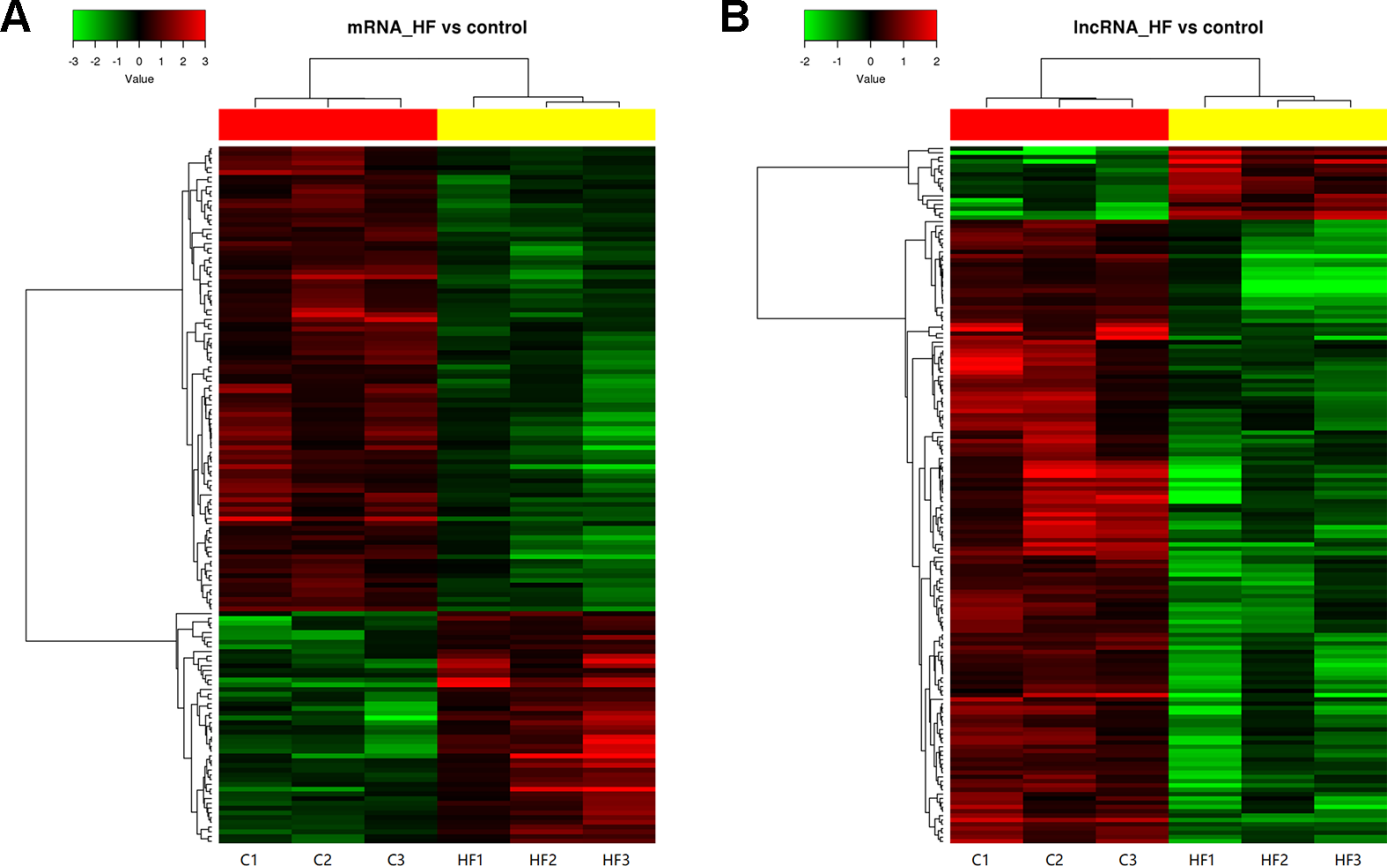
Heat map and hierarchical clustering of messenger RNA (mRNA) **(A)** and long non-coding RNA (lncRNA) **(B)** expression profiles in heart failure compared with controls. Each column represented one sample, and each row represented one mRNA or lncRNA. The relative expression levels of mRNAs and lncRNAs were depicted according to the color scale. Red indicated up-regulation and green indicated down-regulation. 2.0 and −2.0 were fold changes in the corresponding spectrum, whereas left three columns represented control samples and right three columns represented HF samples. The differentially expressed mRNAs and lncRNAs were clearly self-segregated into clusters.

Cluster analyses were performed to show the expression patterns of mRNAs and lncRNAs. Heat maps revealed that hierarchical clustering of the expression of the 147 mRNAs and the 162 lncRNAs based on centered Pearson correlation clearly separated HF from control group (**Figure 4**).

### Validation of the Microarray Data by qPCR

**Figure 5:**
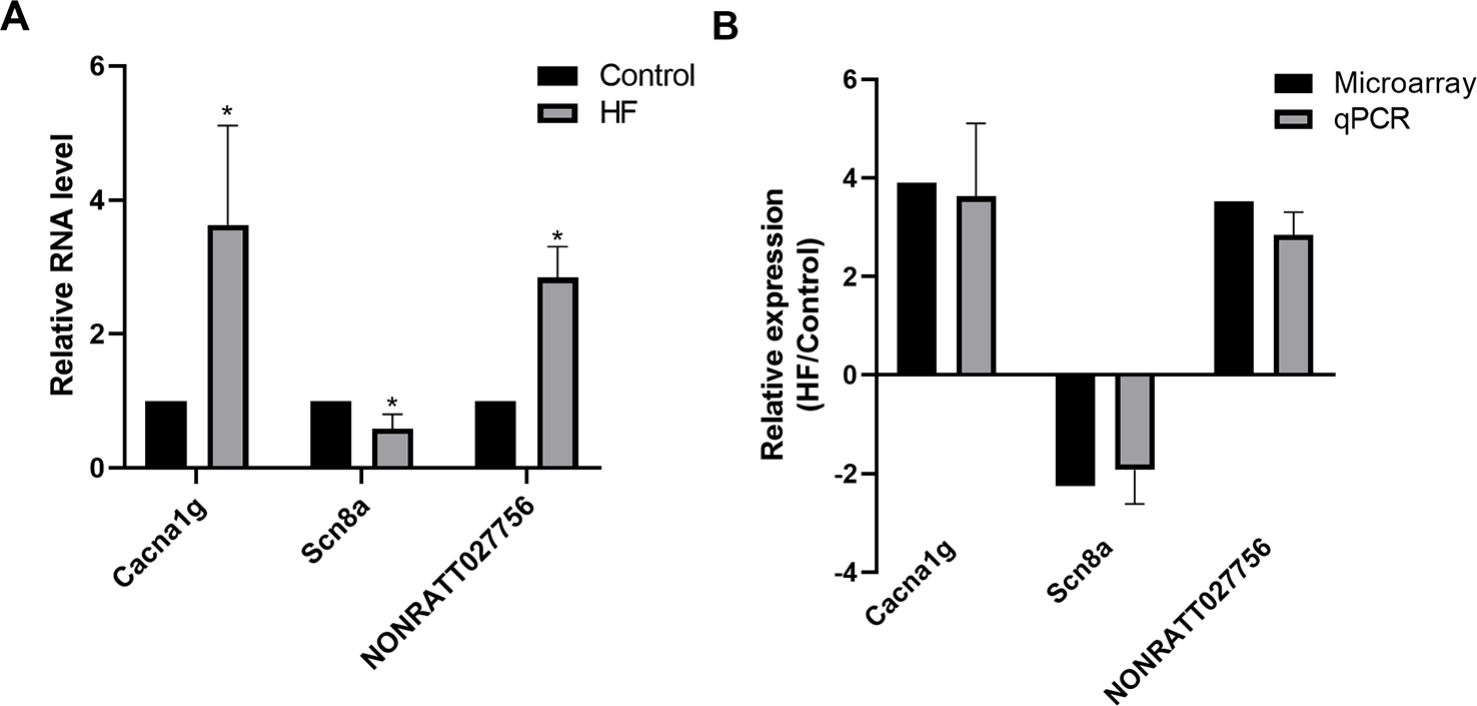
Quantitative PCR (qPCR) validation. **(A)** Relative expression levels of selected messenger RNAs (mRNAs) (Cacna1g, Scn8a) and long non-coding RNA (lncRNA) (NONRATT027756) in heart failure (HF) rat hearts (n = 3) compared with controls (n = 3). **(B)** Comparison between microarray and qPCR results of selected differentially expressed mRNAs (Cacna1g, Scn8a) and lncRNA (NONRATT027756). The qPCR analysis results were consistent with the microarray data. All reactions were repeated three times for each mRNA or lncRNA. Actin was used as internal control. *P < 0.05 HF *vs.* control.

Several significantly differentially expressed mRNAs and lncRNAs of interest for further analysis were selected to prove the stability and precision of the microarray expression data by qPCR, including one up-regulated mRNA (Cacna1g), one down-regulated mRNA (Scn8a), and one up-regulated lncRNA (NONRATT027756). Verification results confirmed that the expression of Cacna1g was up-regulated and Scn8a was down-regulated, meanwhile NONRATT027756 was significantly up-regulated in the HF hearts compared with controls (**Figure 5A**), consistent with the microarray analysis results (**Figure 5B**). Therefore, the qPCR results supported the accuracy and reliability of the microarray analysis.

### Gene Ontology and Kyoto Encyclopedia of Genes and Genomes Pathway Analysis

To predict the potential functions of the dysregulated mRNAs in HF, these mRNAs were detected by enrichment analysis, including GO analysis, KEGG, and PANTHER Pathway analyses.

**Figure 6:**
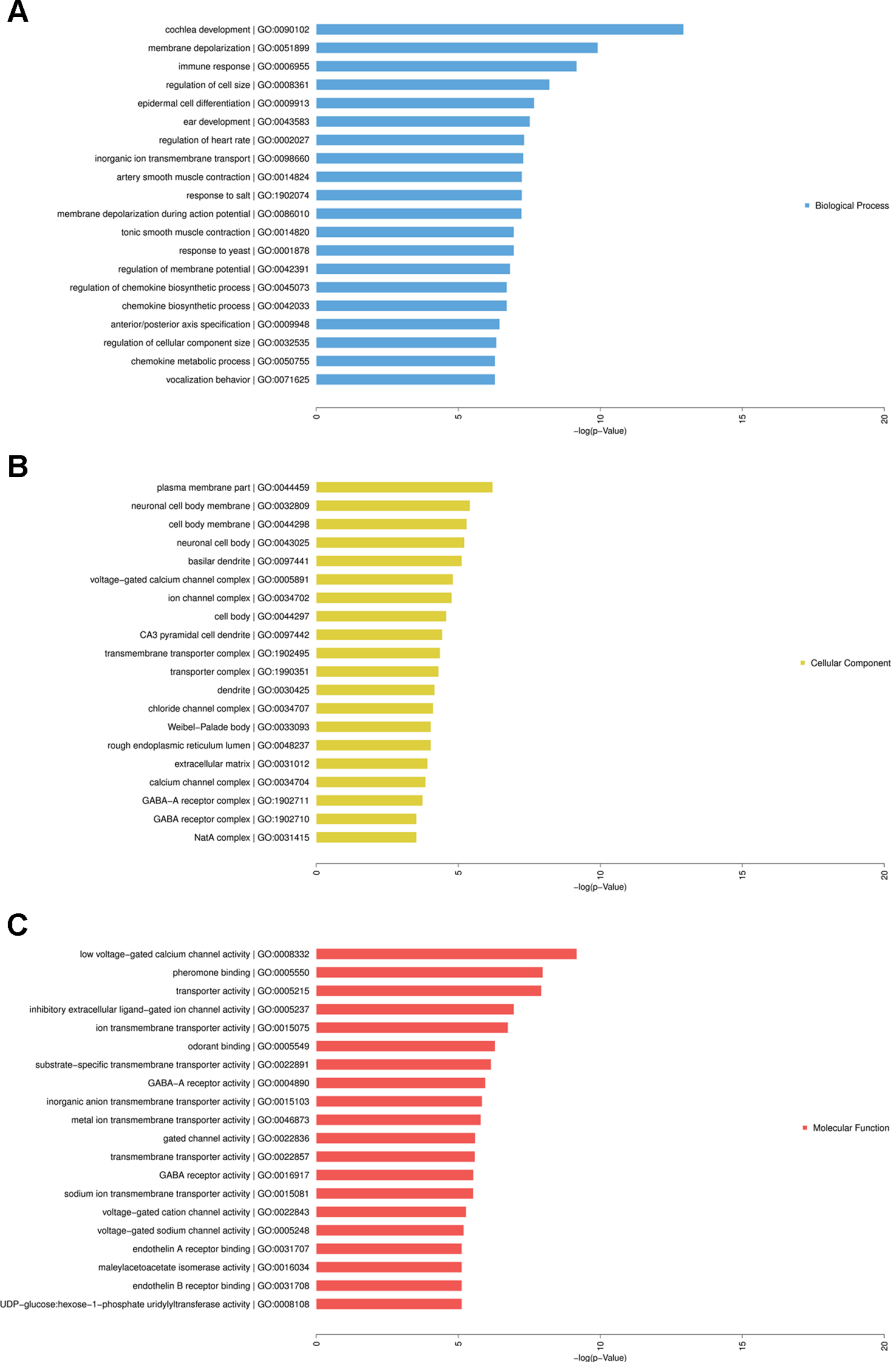
Gene Ontology (GO) analysis of differentially expressed messenger RNAs (mRNAs) in heart failure. Enriched mRNAs were functionally classified by biological process (BP), cellular component (CP), and molecular function (MF). Top 20 significantly enriched GO terms of BP **(A)**, CP **(B)**, and MF **(C)** were listed.

GO analysis showed that complicated functional pathways were enriched in the HF group compared with the controls, which contained associated differentially expressed mRNAs with *P*-value < 0.05. The top 20 significantly enriched GO terms were shown in **Figure 6**. These enriched mRNAs were functionally classified by BP (**Figure 6A**), CC (**Figure 6B**), and MF (**Figure 6C**). Among them, several BPs seemed to be associated with the mechanisms of HF, for instance, regulation of cell size, epidermal cell differentiation, immune response, regulation of heart rate, smooth muscle contraction. Moreover, many CCs and MFs were found to be related to regulation of cardiac electrical activity, such as membrane depolarization, ion transmembrane transport, regulation of membrane potential, ion channel complex, ion transmembrane transporter activity.

**Figure 7:**
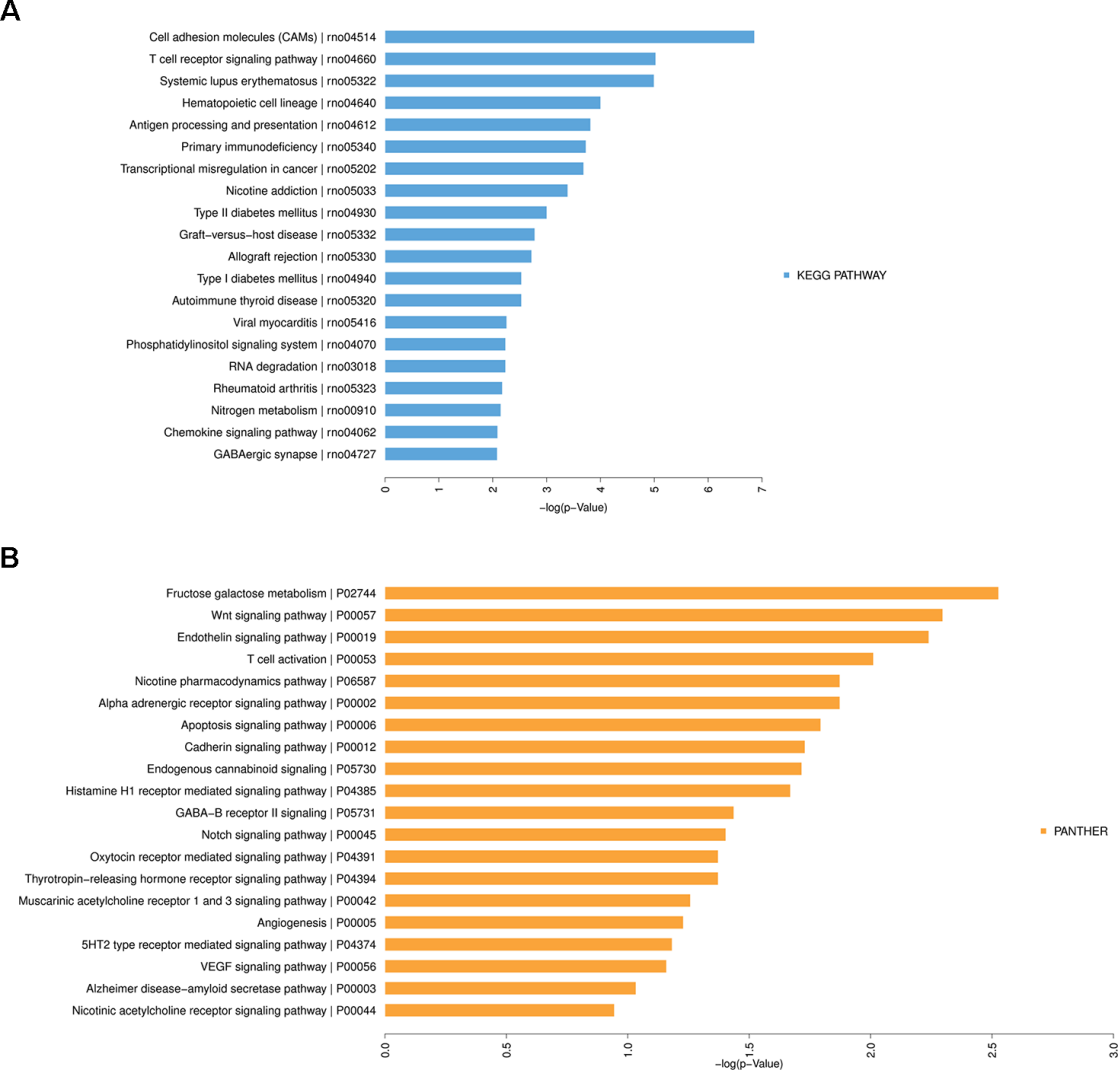
Kyoto Encyclopedia of Genes and Genomes (KEGG) Pathway analysis of differentially expressed messenger RNAs in heart failure. **(A)** Top 20 significantly enriched KEGG Pathway terms. **(B)** Top 20 significantly enriched PANTHER Pathway terms.

Furthermore, KEGG and PANTHER Pathway analyses indicated that several significantly enriched pathways were associated with HF. The top 20 enriched KEGG (**Figure 7A**) and PANTHER (**Figure 7B**) pathway terms were listed in **Figure 7**, including cell adhesion molecules, T cell receptor signaling pathway, fructose galactose metabolism, Wnt signaling pathway, endothelin signaling pathway, etc. More details about these enriched terms or pathways and contained genes were shown in **Tables S2–S6**.

### Construction of mRNA-lncRNA Co-Expression Network

To explore the role and interaction of genes in HF, the mRNA-lncRNA co-expression network was constructed based on the correlation analysis between the differentially expressed mRNAs and lncRNAs. The Pearson’s correlation coefficients were calculated for each pair of genes and significantly correlated pairs of mRNAs and lncRNAs with Pearson’s correlation no less than 0.99 were chosen to build the network.

**Figure 8:**
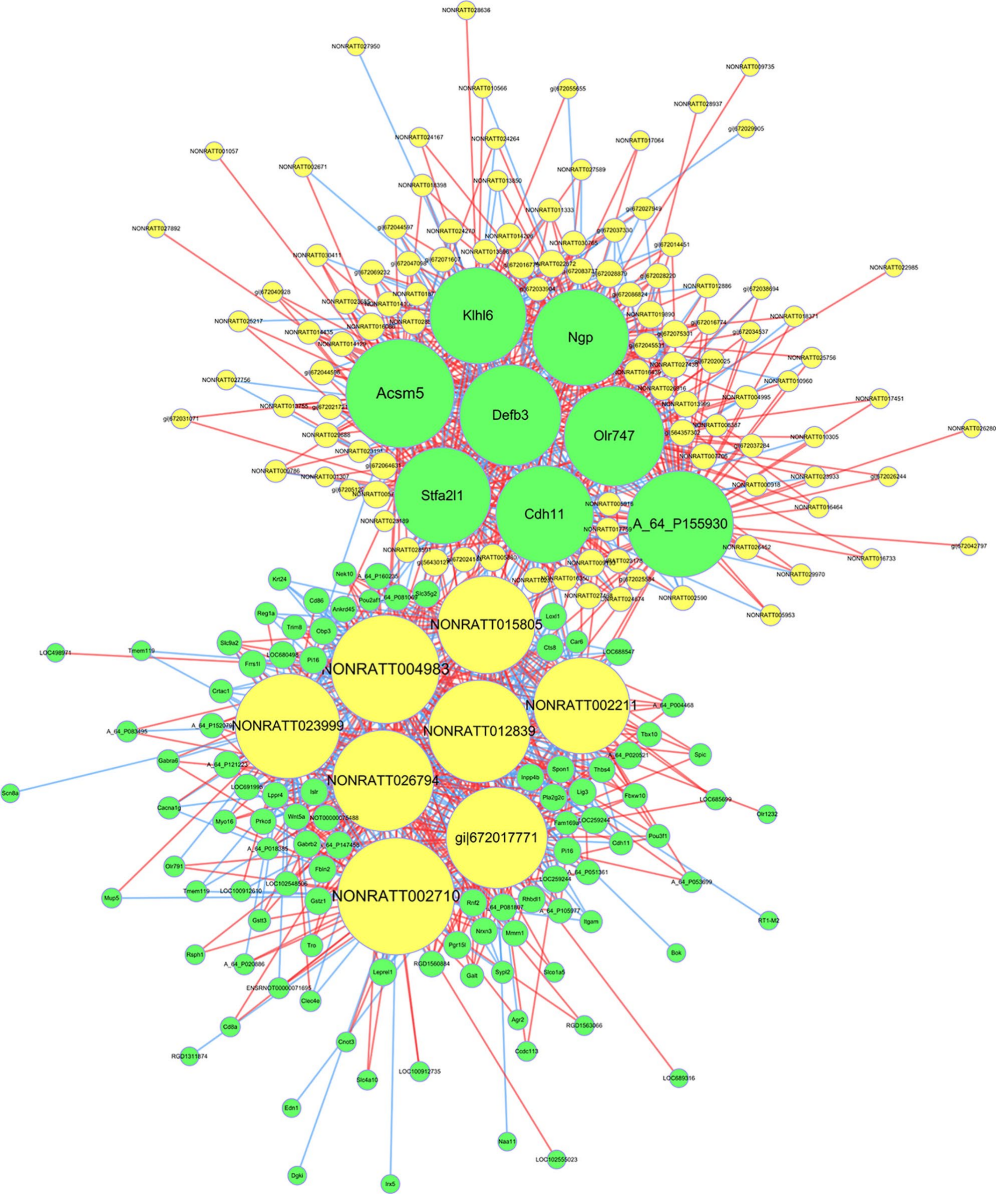
The messenger RNA (mRNA)-long non-coding RNA (lncRNA) co-expression network in heart failure. The network displayed the correlations between the differentially expressed mRNA and lncRNA profiles. The yellow nodes represented the lncRNAs, and green nodes represented the mRNAs. The size of node represented the degree centrality of the gene in the network, defined as the link numbers of the node. The red lines indicated a positive correlation, while blue lines indicated a negative correlation.

As shown in the network analysis (**Figure 8**), eight mRNAs (Acsm5, A_64_P155930, Defb3, Olr747, Klhl6, Ngp, Stfa2l1, Cdh11) and eight lncRNAs (NONRATT002710, NONRATT004983, NONRATT023999, NONRATT012839, NONRATT026794, NONRATT015805, NONRATT002211, XR_345427.2) were identified as central genes with a relatively higher degree centrality. These genes were suggested to play critical roles in the co-expression network.

### Target Prediction and Transcription Factor Prediction of lncRNAs

**Figure 9:**
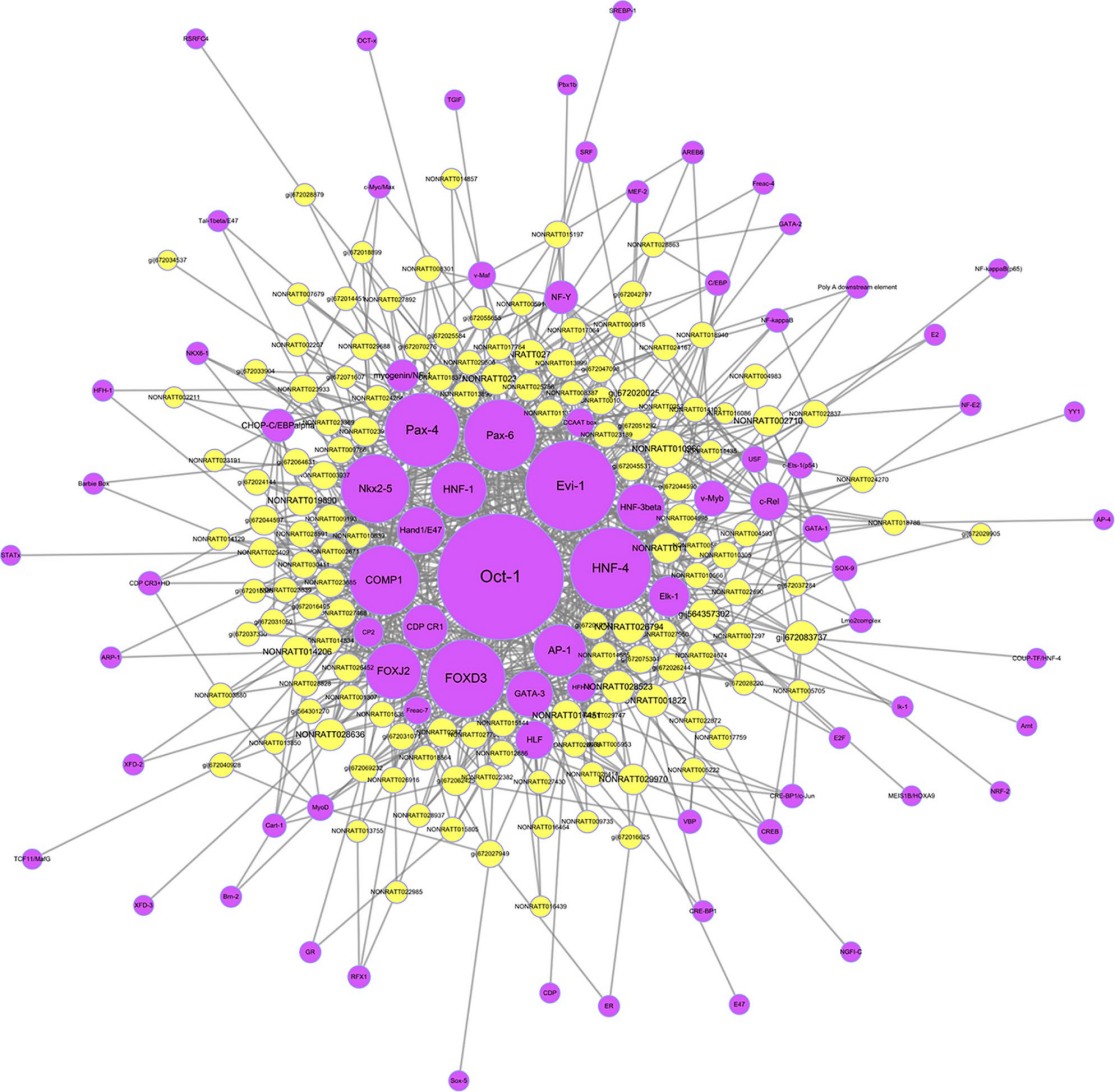
The transcription factors (TFs)-long non-coding RNA (lncRNA) network in heart failure. The network was built on the base of interaction between the lncRNAs and TFs. The purple nodes represented TFs, and yellow nodes represented lncRNAs.

To further clarify the potential functions and regulatory mechanisms of these differentially expressed lncRNAs, target prediction of lncRNAs including *cis*-regulation and *trans*-regulation were performed. The most closely related lncRNAs and their predicted *cis*-regulated and *trans*-regulated protein-coding genes were identified. The lncRNA NONRATT013999 was predicted to *cis*-regulate mRNA CDH11; meanwhile, NONRATT027756 was predicted to *trans*-regulate HCN4. The expression levels of these lncRNAs and their target mRNAs were simultaneously up-regulated in HF. Moreover, TFs significantly correlated with lncRNAs were predicted and a TFs-lncRNA co-expression network was built to explore potential regulatory functions of lncRNAs. As shown in **Figure 9**, most of lncRNAs participated in pathways regulated by several crucial TFs, including Oct-1, Evi-1, FOXD3, HNF-4, Pax-4, Pax-6, Nkx2-5, COMP1, and so on. These network analyses might provide certain references for further research.

## Discussion

HF is the complex syndrome as a result of re-expression of comprehensive genes and protein synthesis (Tham et al., 2015; Kim et al., 2018). The researches and therapy methods of HF have already developed for several decades, however, the exact pathogenesis particularly related to transcriptional or post-transcriptional regulation is still not well elucidated (Creemers et al., 2011; Di Salvo and Haldar, 2014; Smith, 2017). Cardiac hypertrophy caused by hypertension is one of the most important pathogenic mechanisms of HF (Oka et al., 2014), especially heart failure with preserved ejection fraction (HFpEF) (Tam et al., 2017), an important type of HF with unclear mechanisms and few effective treatments (Borlaug and Paulus, 2011). Although lncRNAs have been identified as important regulatory factors in the pathophysiological mechanisms of cardiovascular diseases (Ounzain et al., 2015; Leimena and Qiu, 2018; Li et al., 2018), there is scarcely any detailed mRNAs and lncRNAs co-expression analysis of pressure overload-induced HF. Therefore, this study was designed to discover the comprehensive characteristics of the expression profiles of mRNAs and lncRNAs in HF, the interaction and co-expression network of mRNAs and lncRNAs, and the regulatory function prediction of lncRNAs.

Our study investigated the expression profiles of mRNAs and lncRNAs in the rat pressure overload-induced HF model and indicated that 147 mRNAs and 166 lncRNAs displayed significantly differential expression in HF compared with controls. The mRNA-lncRNA and TFs-lncRNA networks were constructed to predict the potential functions of lncRNAs in HF. Although most of these differentially expressed mRNAs and lncRNAs had not been functionally characterized, this study provided a comprehensive understanding of mRNA and lncRNA expression profiles regulation in HF and provided evidences for us to further elucidate the complex regulatory mechanisms underlying HF.

To further precisely clarify these differentially expressed mRNA genes, the GO and Pathway analyses were used to explore the potential biological pathways and functions. The GO analysis results revealed that the most enriched BPs included membrane depolarization, immune response, regulation of cell size, regulation of heart rate, inorganic ion transmembrane transport, artery smooth muscle contraction, response to salt. These BPs or CCs and MFs seemed to be mainly associated with the mechanisms of hypertension, cardiac hypertrophy, arrhythmia, and HF. HF involves diverse pathophysiologic processes in the failing heart, such as cardiomyocyte hypertrophy, cardiomyocyte proliferation, myocardial fibrosis, cardiac electrical remodeling, energetic metabolism, inflammation, and immune response (Chung et al., 2013; Segura et al., 2014; D’Onofrio et al., 2016; Zhou and Tian, 2018). Similar results were obtained by the KEGG and PANTHER Pathway analyses. Particularly, most of these enriched pathway terms might be related to the immune and inflammation response, including cell adhesion molecules, T cell activation, T cell receptor signaling pathway, antigen processing, and presentation. It indicated that more attentions needed to be focused on the inflammation and immune response in pathogenesis of cardiac remodeling and HF. These results were consistent with previous researches (Zhang et al., 2017; Michels da Silva et al., 2019), which had also found that elevated levels of inflammatory cytokines were associated with the severity and prognosis of HF (Michels da Silva et al., 2019). In consequence, our microarray results provided more potential pathogenic or protective genes involved in HF and further confirmed the key roles of these pathways in mechanisms of HF.

Furthermore, this study was aimed to explore the relationship between lncRNA expression profiles and cardiac remodeling and dysfunction in HF. According to previous studies, lncRNA expression profiles were significantly dysregulated in ischemic heart disease (Greco et al., 2016), demonstrating their potential as biomarkers of cardiac remodeling (Kumarswamy et al., 2014) and to regulate cellular functions in HF (Boeckel et al., 2019). Our data further suggested that lncRNAs played important roles in the molecular mechanisms of HF and involved in the regulation of diverse cellular processes. Based on the microarray data, a co-expression network of mRNAs and lncRNAs was constructed to reflect the interactions between genes. Our results found that several lncRNAs acted as regulatory network nodes of mRNAs, which were more closely related to specific mRNAs. However, it still needs more investigations to confirm the relationship between lncRNAs and regulated mRNAs, and the certain functions of these lncRNAs during the development of HF.

Up to now, functions of most lncRNAs have not been clearly illustrated. Therefore, we performed target gene prediction based on *cis*- and *trans*-regulation. Our results showed that an unannotated lncRNA NONRATT013999 was predicted to *cis*-regulate the expression of mRNA CDH11 by interacting with nearby protein-coding genes. Cadherin-11 (CDH11) is an adhesion protein mainly expressed in mesenchymal tissues that regulates the differentiation and function of osteoblasts. CDH11 was identified to participate in multiple BPs including bone formation, cellular signal transduction, tumor invasion, and metastasis (Lee et al., 2007; Li et al., 2012; Langhe et al., 2016; Madarampalli et al., 2019; Yuan et al., 2019a), and also play important roles in developmental and pathogenic mechanisms of heart valve (Zhou et al., 2013; Bowler et al., 2018). A recent study found that CDH11 contributed to inflammation-driven fibrotic remodeling after myocardial infarction (Schroer et al., 2019), which indicated that the increased CDH11 might be involved in the development of HF through inflammatory response. Besides, lncRNA NONRATT027756 was predicted to *trans*-regulate HCN4 by means of miRNA sequestration. Hyperpolarization activated cyclic nucleotide gated potassium channel 4 (HCN4) shows slow kinetics of activation and inactivation, and is necessary in cardiac pacemaking and conduction processes (Liang et al., 2015; Verkerk and Wilders, 2015). Abnormalities of HCN4 may result in a variety of arrhythmias, such as atrial fibrillation, sick sinus syndrome (DiFrancesco, 2015; Ishikawa et al., 2017). Increased HCN4 expression was reported in failing hearts in previous studies, which was consistent with our results. HCN4 overexpression mediated I_f_ augmentation in cardiomyocytes and led to cardiac remodeling (Yampolsky et al., 2019). Although functions of NONRATT013999 and NONRATT027756 were still unclear, the expression levels of these lncRNAs were increased in HF. They might up-regulate their target mRNAs, CDH11 and HCN4, further suggested their potential roles in the mechanism of HF. In addition, prediction of regulatory TFs was performed and TFs-lncRNA network was constructed in the current study. Shown in the network, several TFs were identified to play vital roles in regulating the function of lncRNAs, including Oct-1, Evi-1, FOXD3, HNF-4, Pax-4, Pax-6, Nkx2-5, COMP1. The interaction between lncRNAs and the TFs mentioned above needs to be explored more deeply. Therefore, further functional verification studies and analyses of lncRNAs should be conducted to elucidate the precise transcriptional and post-transcriptional regulatory network in HF.

HF is a clinical syndrome that is heterogeneous in both pathophysiology and etiology. In summary, we provided a comprehensive understanding of mRNA and lncRNA expression profiles regulation in pressure overload-induced HF rats. We found that the lncRNA NONRATT013999 was predicted to *cis-*regulate mRNA CDH11, and NONRATT027756 was predicted to *trans-*regulate HCN4. Whether these changes also occurred in other HF models or clinical samples we didn’t investigate in this study. Moreover, the functions of lncRNAs and their interactions with mRNAs in HF were only preliminary predicted using bioinformatics analyses. Therefore, further researches with more HF samples are required and validation experiments need to be conducted *in vivo* and *in vitro* to clarify our findings.

## Conclusion

In conclusion, we explored the comprehensive characteristics of mRNAs and lncRNAs in pressure overload-induced HF. The mRNA and lncRNA expression profiles were significantly dysregulated in the HF rat hearts compared with controls. Variety of differentially expressed mRNAs were involved in diverse biological pathways related to the mechanisms of cardiac hypertrophy and HF. Through the co-expression network, complex interactions between mRNAs and lncRNAs were demonstrated and several lncRNAs were identified as key regulating elements. Several target mRNAs and regulatory TFs were identified by *cis*- and *trans*-regulation prediction of lncRNAs. Based on the present study, further investigations should be required to clarify the specific functions of these mRNAs and lncRNAs in the pathogenesis of HF.

## Data Availability Statement

All datasets generated for this study are included in the article/**Supplementary Material**.

## Ethics Statement

The animal study was reviewed and approved by the Ethical Committee of Xi’an Jiaotong University.

## Author Contributions

LB and AM conceived the study. SC, QM, YX, JZ, GY performed the experiments. SC and TW analyzed the data. SC wrote the manuscript. TW, AM, and LB reviewed and revised the manuscript.

## Funding

This study was supported by the National Natural Science Foundation of China (No. 81270235) and the Science and Technology Project of Shaanxi Province (No. 2012K15-01-01).

## Conflict of Interest

The authors declare that the research was conducted in the absence of any commercial or financial relationships that could be construed as a potential conflict of interest.
