# Supplementary material for: Comprehensive Analysis and Co-Expression Network of mRNAs and lncRNAs in Pressure Overload-Induced Heart Failure
Source: Front Genet. 2019 Dec 12;10:1271. doi: 10.3389/fgene.2019.01271 (PMC6920101; doi:10.3389/fgene.2019.01271)
Supplement: Supplementary file 1 [file DataSheet_1.docx]

Supplementary Material

**Table S1. Characteristics of the SO rats and pressure-overload HF rats at 16 weeks after TAC.**

| **Group** | **SO (n=10)** | **HF (n=10)** |
| --- | --- | --- |
| Echocardiography |  |  |
| LVEDd (mm) | 6.76±0.64 | 7.00±0.55 |
| LVEDs (mm) | 3.83±0.56 | 4.22±0.32 |
| LVWTd (mm) | 1.44±0.12 | 1.75±0.28^*^ |
| LVWTs (mm) | 2.19±0.19 | 2.57±0.36^*^ |
| IVSTd (mm) | 1.40±0.20 | 1.64±0.27^*^ |
| IVSTs (mm) | 1.94±0.28 | 2.33±0.35^*^ |
| LVEDV (ml) | 0.719±0.205 | 0.785±0.175 |
| LVESV (ml) | 0.161±0.068 | 0.189±0.039 |
| LVEF (%) | 80.0±3.7 | 75.8±3.3^*^ |
| FS (%) | 43.6±3.4 | 39.7±2.7^*^ |
| HR (bpm) | 379±42 | 375±37 |
| HW/BW (g/g) | 0.295±0.044 | 0.395±0.037^*^ |

Values are mean±SD; n, the number of rats.

LVEDd: left ventricular end-diastolic diameter; LVEDs: left ventricular end-systolic diameter; LVWTd: left ventricular wall diastolic thickness; LVWTs: left ventricular wall systolic thickness; IVSTd: interventricular septum diastolic thickness; IVSTs: interventricular septum systolic thickness; LVEDV: left ventricular end-diastolic volume; LVESV: left ventricular end-systolic volume; LVEF: left ventricular ejection fraction; FS: fractional shortening; HR: heart rate; HW/BW: heart weight/body weight ratio.

* *P* < 0.05 HF *vs.* SO.

**Table S2. Top 20 enriched pathways of biological process associated with HF via GO analysis.**

| **Term** | **ID** | ***P*-value** | **Genes** |
| --- | --- | --- | --- |
| cochlea development | GO: 0090102 | 2.411623e-06 | Gabrb2: down; Hey2: down; Slc26a5: up; Atp2b2: up; Wnt5a: down |
| membrane depolarization | GO: 0051899 | 4.928045e-05 | Scn8a: down; Cacna1g: up; Cacna1h: up; Dgki: up; Edn1: up; Hcn4: up |
| immune response | GO: 0006955 | 1.037656e-04 | Cd8a: down; Elane: down; Wnt5a: down; Edn1: up; Clec4e: up; Krt16: down; Mcpt8l2: down; Prkcd: up; Itk: down; Cd86: up; Trim8: down; Cd247: down; Klhl6: up; RT1-M2: up |
| regulation of cell size | GO: 0008361 | 2.702350e-04 | Slc26a5: up; Atp2b2: upWnt5a: down; Edn1: up; Dscam: down |
| epidermal cell differentiation | GO: 0009913 | 4.635426e-04 | Krt16: down; Hey2: down; Atp2b2: up; Wnt5a: down; Pou3f1: down |
| ear development | GO: 0043583 | 5.385180e-04 | Gabrb2: down; Hey2: down; Slc26a5: up; Atp2b2: up; Wnt5a: down; Edn1: up |
| regulation of heart rate | GO: 0002027 | 6.582625e-04 | Hey2: down; Cacna1g: up; Edn1: up; Hcn4: up |
| inorganic ion transmembrane transport | GO: 0098660 | 6.786015e-04 | Slc4a10: down; Cacna1g: up; Cacna1h: up; Slc26a5: up; Atp2b2: up; Slc9a2: down; Gabra6: down; Hcn4: up; Scn8a: down; Gabrb2: down |
| artery smooth muscle contraction | GO: 0014824 | 7.131124e-04 | Cacna1g: up; Edn1: up |
| response to salt | GO: 1902074 | 7.131124e-04 | Slc26a5: up; Edn1: up |
| membrane depolarization during action potential | GO: 0086010 | 7.232204e-04 | Scn8a: down; Cacna1g: up; Cacna1h: up |
| tonic smooth muscle contraction | GO: 0014820 | 9.471638e-04 | Cacna1g: up; Edn1: up |
| response to yeast | GO: 0001878 | 9.471638e-04 | Cd86: up; Elane: down |
| regulation of membrane potential | GO: 0042391 | 1.080257e-03 | Scn8a: down; Cacna1g: up; Cacna1h: up; Slc26a5: up; Dgki: up; Edn1: up; Hcn4: up |
| regulation of chemokine biosynthetic process | GO: 0045073 | 1.213106e-03 | Elane: down; Wnt5a: down |
| chemokine biosynthetic process | GO: 0042033 | 1.213106e-03 | Elane: down; Wnt5a: down |
| anterior/posterior axis specification | GO: 0009948 | 1.566798e-03 | Hey2: down; Wnt5a: down; Rnf2: down |
| regulation of cellular component size | GO: 0032535 | 1.751281e-03 | Prkcd: up; Slc26a5: up; Atp2b2: up; Wnt5a: down; Edn1: up; Dscam: down |
| chemokine metabolic process | GO: 0050755 | 1.839159e-03 | Elane: down; Wnt5a: down |
| vocalization behavior | GO: 0071625 | 1.839159e-03 | Celf6: down; Nrxn3: down |

**Table S3. Top 20 enriched pathways of cellular component associated with HF via GO analysis.**

| **Term** | **ID** | ***P*-value** | **Genes** |
| --- | --- | --- | --- |
| plasma membrane part | GO: 0044459 | 2.000273e-03 | Cacna1g: upCacna1h: up; Cd8a: down; Slc26a5: up; Slc9a2: down; Scn8a: down; Gabrb2: down; Dgki: up; Slc4a10: down; Atp2b2: up; Slco1a5: down; Cd8b: down; Hcn4: up; Gabra6: down; Itk: down; Cd86: up; Cd247: down; RT1-M2: up |
| neuronal cell body membrane | GO: 0032809 | 4.444205e-03 | Atp2b2: up; Gabra6: down |
| cell body membrane | GO: 0044298 | 4.980607e-03 | Atp2b2: up; Gabra6: down |
| neuronal cell body | GO: 0043025 | 5.415474e-03 | Slc4a10: down; Cacna1g: up; Cacna1h: up; Atp2b2: up; Gabra6: down; Hcn4: up; Scn8a: down; Dgki: up |
| basilar dendrite | GO: 0097441 | 5.920187e-03 | Slc4a10: down |
| voltage-gated calcium channel complex | GO: 0005891 | 8.079800e-03 | Cacna1g: up; Cacna1h: up |
| ion channel complex | GO: 0034702 | 8.423466e-03 | Scn8a: down; Gabrb2: down; Cacna1g: up; Cacna1h: up; Gabra6: down |
| cell body | GO: 0044297 | 1.024084e-02 | Slc4a10: down; Cacna1g: up; Cacna1h: up; Atp2b2: up; Gabra6: down; Hcn4: up; Scn8a: down; Dgki: up |
| CA3 pyramidal cell dendrite | GO: 0097442 | 1.180576e-02 | Slc4a10: down |
| transmembrane transporter complex | GO: 1902495 | 1.275626e-02 | Scn8a: down; Gabrb2: down; Cacna1g: up; Cacna1h: up; Gabra6: down |
| transporter complex | GO: 1990351 | 1.341479e-02 | Scn8a: down; Gabrb2: down; Cacna1g: up; Cacna1h: up; Gabra6: down |
| dendrite | GO: 0030425 | 1.545834e-02 | Scn8a: down; Slc4a10: down; Cacna1g: up; Cacna1h: up; Atp2b2: up; Dgki: up; Gabra6: down |
| chloride channel complex | GO: 0034707 | 1.623109e-02 | Gabrb2: down; Gabra6: down |
| Weibel-Palade body | GO: 0033093 | 1.765691e-02 | Edn1: up |
| rough endoplasmic reticulum lumen | GO: 0048237 | 1.765691e-02 | Edn1: up |
| extracellular matrix | GO: 0031012 | 1.981405e-02 | Fbln2: up; Loxl1: up; Wnt5a: down; P3h2: up; Spon1: up |
| calcium channel complex | GO: 0034704 | 2.120381e-02 | Cacna1g: up; Cacna1h: up |
| GABA-A receptor complex | GO: 1902711 | 2.347384e-02 | Gabrb2: down |
| GABA receptor complex | GO: 1902710 | 2.925674e-02 | Gabrb2: down |
| NatA complex | GO: 0031415 | 2.925674e-02 | Naa11: up |

**Table S4. Top 20 enriched pathways of molecular function associated with HF via GO analysis.**

| **Term** | **ID** | ***P*-value** | **Genes** |
| --- | --- | --- | --- |
| low voltage-gated calcium channel activity | GO: 0008332 | 1.034555e-04 | Cacna1g: up; Cacna1h: up |
| pheromone binding | GO: 0005550 | 3.422032e-04 | Obp3: up; rno: 259244: up |
| transporter activity | GO: 0005215 | 3.602492e-04 | Slc4a10: down; Cacna1g: up; Cacna1h: up; Obp3: up; Slc26a5: up; Atp2b2: up; Slc9a2: down; Slco1a5: down; Gabra6: down; Hcn4: up; Scn8a: down; Gabrb2: down; Sypl2: up; rno: 259244: up; Rph3a: down |
| inhibitory extracellular ligand-gated ion channel activity | GO: 0005237 | 9.471638e-04 | Gabrb2: down; Gabra6: down |
| ion transmembrane transporter activity | GO: 0015075 | 1.165336e-03 | Slc4a10: down; Cacna1g: up; Cacna1h: up; Slc26a5: up; Atp2b2: up; Slc9a2: down; Slco1a5: down; Gabra6: down; Hcn4: up; Scn8a: down; Gabrb2: down |
| odorant binding | GO: 0005549 | 1.839159e-03 | Obp3: up; rno: 259244: up |
| substrate-specific transmembrane transporter activity | GO: 0022891 | 2.115207e-03 | Slc4a10: down; Cacna1g: up; Cacna1h: up; Slc26a5: up; Atp2b2: up; Slc9a2: down; Slco1a5: down; Gabra6: down; Hcn4: up; Scn8a: down; Gabrb2: down |
| GABA-A receptor activity | GO: 0004890 | 2.588299e-03 | Gabrb2: down; Gabra6: down |
| inorganic anion transmembrane transporter activity | GO: 0015103 | 2.906247e-03 | Gabrb2: down; Slc4a10: down; Slc26a5: up; Gabra6: down |
| metal ion transmembrane transporter activity | GO: 0046873 | 3.040634e-03 | Scn8a: down; Slc4a10: down; Cacna1g: up; Cacna1h: up; Atp2b2: up; Slc9a2: down; Hcn4: up |
| gated channel activity | GO: 0022836 | 3.699269e-03 | Scn8a: down; Gabrb2: down; Cacna1g: up; Cacna1h: up; Gabra6: down; Hcn4: up |
| transmembrane transporter activity | GO: 0022857 | 3.736301e-03 | Slc4a10: down; Cacna1g: up; Cacna1h: up; Slc26a5: up; Atp2b2: up; Slc9a2: down; Slco1a5: down; Gabra6: down; Hcn4: up; Scn8a: down; Gabrb2: down |
| GABA receptor activity | GO: 0016917 | 3.936421e-03 | Gabrb2: down; Gabra6: down |
| sodium ion transmembrane transporter activity | GO: 0015081 | 3.961353e-03 | Scn8a: down; Slc4a10: down; Slc9a2: down; Hcn4: up |
| voltage-gated cation channel activity | GO: 0022843 | 5.094572e-03 | Scn8a: down; Cacna1g: up; Cacna1h: up; Hcn4: up |
| voltage-gated sodium channel activity | GO: 0005248 | 5.545278e-03 | Scn8a: down; Hcn4: up |
| endothelin A receptor binding | GO: 0031707 | 5.920187e-03 | Edn1: up |
| maleylacetoacetate isomerase activity | GO: 0016034 | 5.920187e-03 | Gstz1: down |
| endothelin B receptor binding | GO: 0031708 | 5.920187e-03 | Edn1: up |
| UDP-glucose: hexose-1-phosphate uridylyltransferase activity | GO: 0008108 | 5.920187e-03 | Galt: down |

**Table S5. Top 20 enriched pathways associated with HF via KEGG analysis.**

| **Term** | **ID** | ***P*-value** | **Genes** |
| --- | --- | --- | --- |
| Cell adhesion molecules (CAMs) | rno04514 | 0.001047165 | Nrxn3: down; Cd86: up; RT1-M2: upCd8a: down; Cd8b: down; Itgam: up |
| T cell receptor signaling pathway | rno04660 | 0.006551306 | Cd247: down; Cd8a: down; Cd8b: down; Itk: down |
| Systemic lupus erythematosus | rno05322 | 0.006765285 | Elane: down; Mcpt8l2: down; Cd86: up; rno: 680498: down |
| Hematopoietic cell lineage | rno04640 | 0.018238437 | Itgam: up; Cd8b: down; Cd8a: down |
| Antigen processing and presentation | rno04612 | 0.021998163 | RT1-M2: up; Cd8b: down; Cd8a: down |
| Primary immunodeficiency | rno05340 | 0.023934183 | Cd8b: down; Cd8a: down |
| Transcriptional misregulation in cancer | rno05202 | 0.025001618 | Elane: down; Itgam: up; Cd86: up; rno: 680498: down |
| Nicotine addiction | rno05033 | 0.033575990 | Gabrb2: down; Gabra6: down |
| Type II diabetes mellitus | rno04930 | 0.049477900 | Prkcd: up; Cacna1g: up |
| Graft-versus-host disease | rno05332 | 0.061911753 | RT1-M2: up; Cd86: up |
| Allograft rejection | rno05330 | 0.065640436 | RT1-M2: up; Cd86: up |
| Type I diabetes mellitus | rno04940 | 0.079250105 | RT1-M2: up; Cd86: up |
| Autoimmune thyroid disease | rno05320 | 0.079250105 | RT1-M2: up; Cd86: up |
| Viral myocarditis | rno05416 | 0.104353233 | RT1-M2: up; Cd86: up |
| Phosphatidylinositol signaling system | rno04070 | 0.106533354 | Dgki: up; Inpp4b: down |
| RNA degradation | rno03018 | 0.106533354 | Cnot3: down; Btg4: down |
| Rheumatoid arthritis | rno05323 | 0.113146236 | Ccl5: down; Cd86: up |
| Nitrogen metabolism | rno00910 | 0.116413392 | Car6: up |
| Chemokine signaling pathway | rno04062 | 0.123485513 | Ccl5: down; Prkcd: up; Itk: down |
| GABAergic synapse | rno04727 | 0.124393732 | Gabrb2: down; Gabra6: down |

**Table S6. Top 20 enriched pathways associated with HF via PANTHER analysis.**

| **Term** | **ID** | ***P*-value** | **Genes** |
| --- | --- | --- | --- |
| Fructose galactose metabolism | P02744 | 0.079907944 | Galt: down |
| Wnt signaling pathway | P00057 | 0.100533898 | Edn1: up; Wnt5a: down; Cdh11: up; Prkcd: up |
| Endothelin signaling pathway | P00019 | 0.106483326 | Edn1: up; Prkcd: up |
| T cell activation | P00053 | 0.133823295 | Cd86: up; Cd247: down |
| Nicotine pharmacodynamics pathway | P06587 | 0.153608970 | Cacna1g: up |
| Alpha adrenergic receptor signaling pathway | P00002 | 0.153608970 | Prkcd: up |
| Apoptosis signaling pathway | P00006 | 0.166260515 | Bok: up; Prkcd: up |
| Cadherin signaling pathway | P00012 | 0.177376992 | Wnt5a: down; Cdh11: up |
| Endogenous cannabinoid signaling | P05730 | 0.179743314 | Cacna1g: up |
| Histamine H1 receptor mediated signaling pathway | P04385 | 0.188279571 | Prkcd: up |
| GABA-B receptor II signaling | P05731 | 0.237716758 | Cacna1g: up |
| Notch signaling pathway | P00045 | 0.245667247 | Hey2: down |
| Oxytocin receptor mediated signaling pathway | P04391 | 0.253537301 | Prkcd: up |
| Thyrotropin-releasing hormone receptor signaling pathway | P04394 | 0.253537301 | Prkcd: up |
| Muscarinic acetylcholine receptor 1 and 3 signaling pathway | P00042 | 0.284228828 | Prkcd: up |
| Angiogenesis | P00005 | 0.292638499 | Wnt5a: down; Prkcd: up |
| 5HT2 type receptor mediated signaling pathway | P04374 | 0.306440808 | Prkcd: up |
| VEGF signaling pathway | P00056 | 0.313695151 | Prkcd: up |
| Alzheimer disease-amyloid secretase pathway | P00003 | 0.355700669 | Prkcd: up |
| Nicotinic acetylcholine receptor signaling pathway | P00044 | 0.388791074 | Myo16: down |
